# Supplementary figures and images for: Mapping mutational fitness effects across the coxsackievirus B3 proteome reveals distinct profiles of mutation tolerability
Source: PLoS Biol. 2024 Jul 16;22(7):e3002709. doi: 10.1371/journal.pbio.3002709 (PMC11251597; doi:10.1371/journal.pbio.3002709)

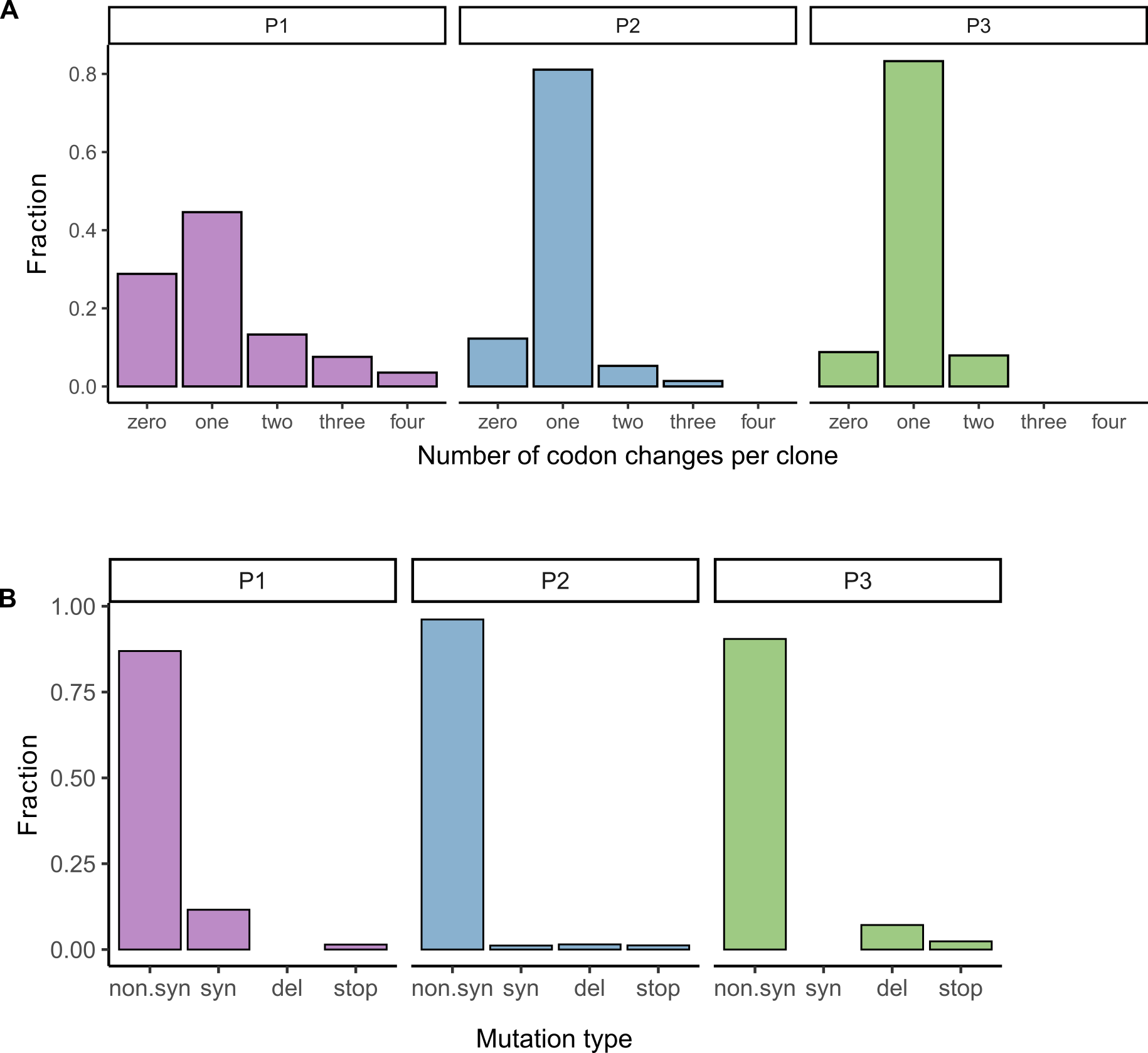

Supplement: S1 Fig — A total of 59, 148, and 92 clones were sequenced for the P1, P2, and P3 region, respectively. The fraction of each number of codons mutated per clone (A) and each type of mutation (B) are graphed. Of note, the full capsid region was sequenced for P1, while for P2 and P3, only the corresponding mutagenized tile region was sequenced. The data underlying this figure can be found in S1 Data, page 9. (TIFF) [file pbio.3002709.s001.tiff]

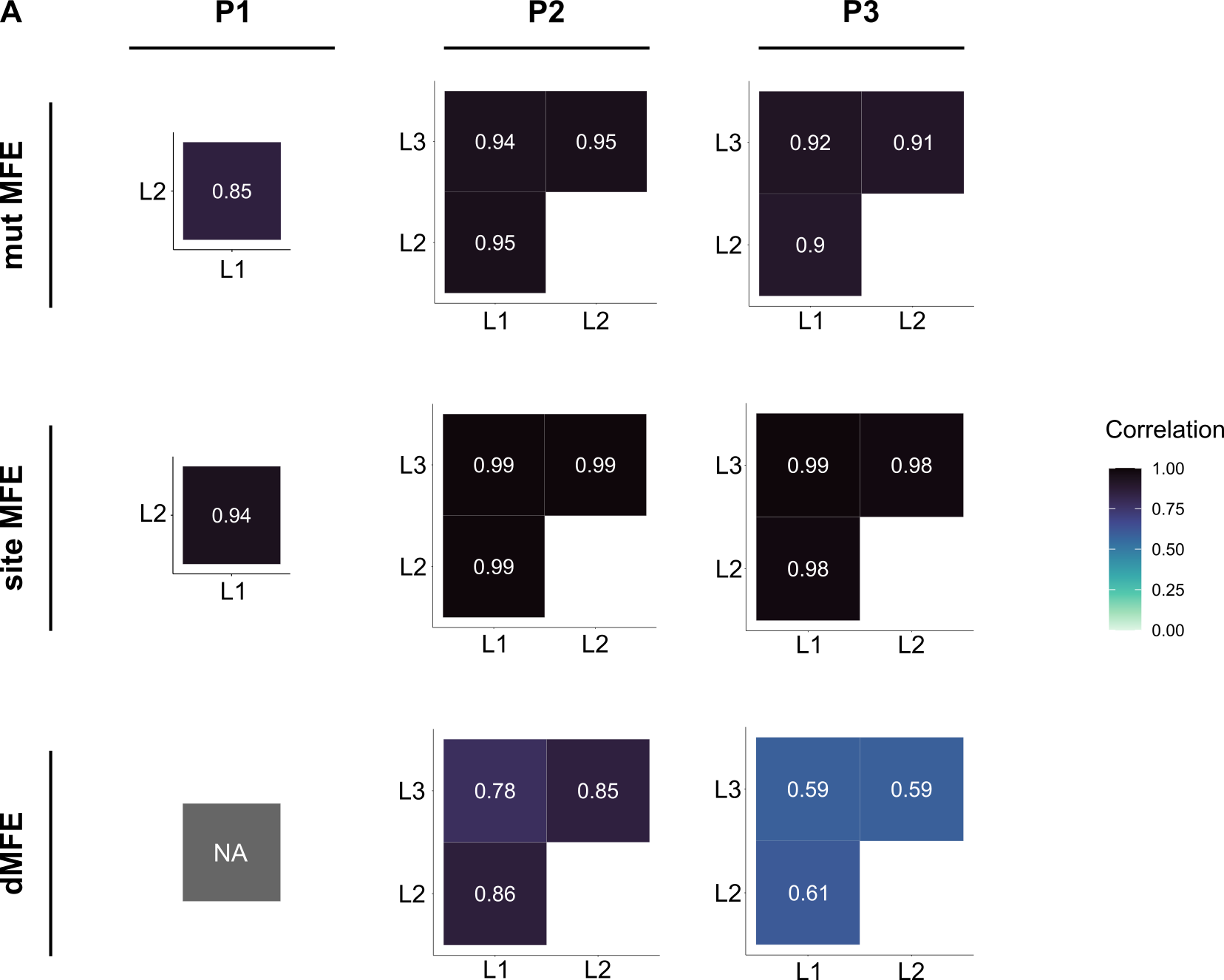

Supplement: S2 Fig — (A) Correlations matrices for MFE of mutations (mut MFE), their average per site (site MFE), and deletions (dMFE) for independent replicate lines (L1-L3) for the P1, P2, and P3 regions. Of note, for P1, only 2 replicates were used and deletions were not included in the mutagenesis protocol, precluding their analysis. (TIFF) [file pbio.3002709.s002.tiff]

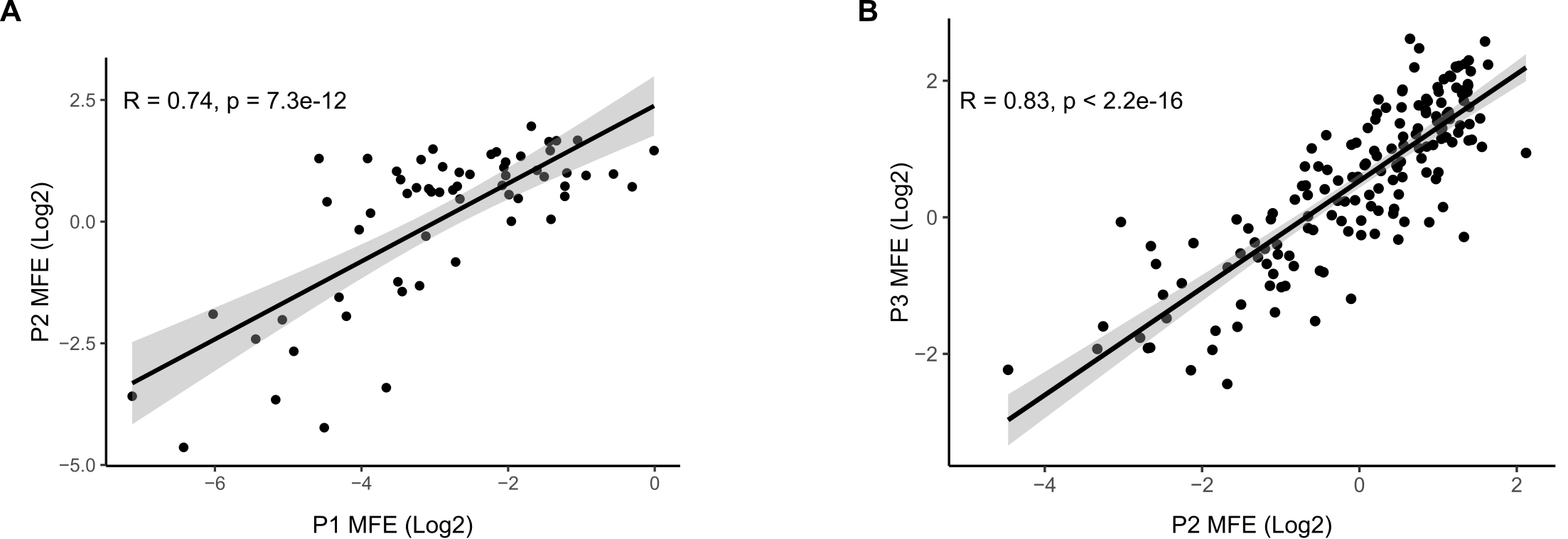

Supplement: S3 Fig — Graphical representation of the linear model (black line) and 95% CI (gray shade) obtained for the MFE of mutations present in the overlap region between P1 and P2 (R2 = 0.54, p = 7.338 × 10^−12^) (A) or P2 and P3 (R2 = 0.69, p < 2.2 × 10^−16^) (B) used for normalization between regions. The Pearson correlation coefficient and associated p-value are shown. The data underlying this figure can be found in S1 Data, page 10. (TIFF) [file pbio.3002709.s003.tiff]

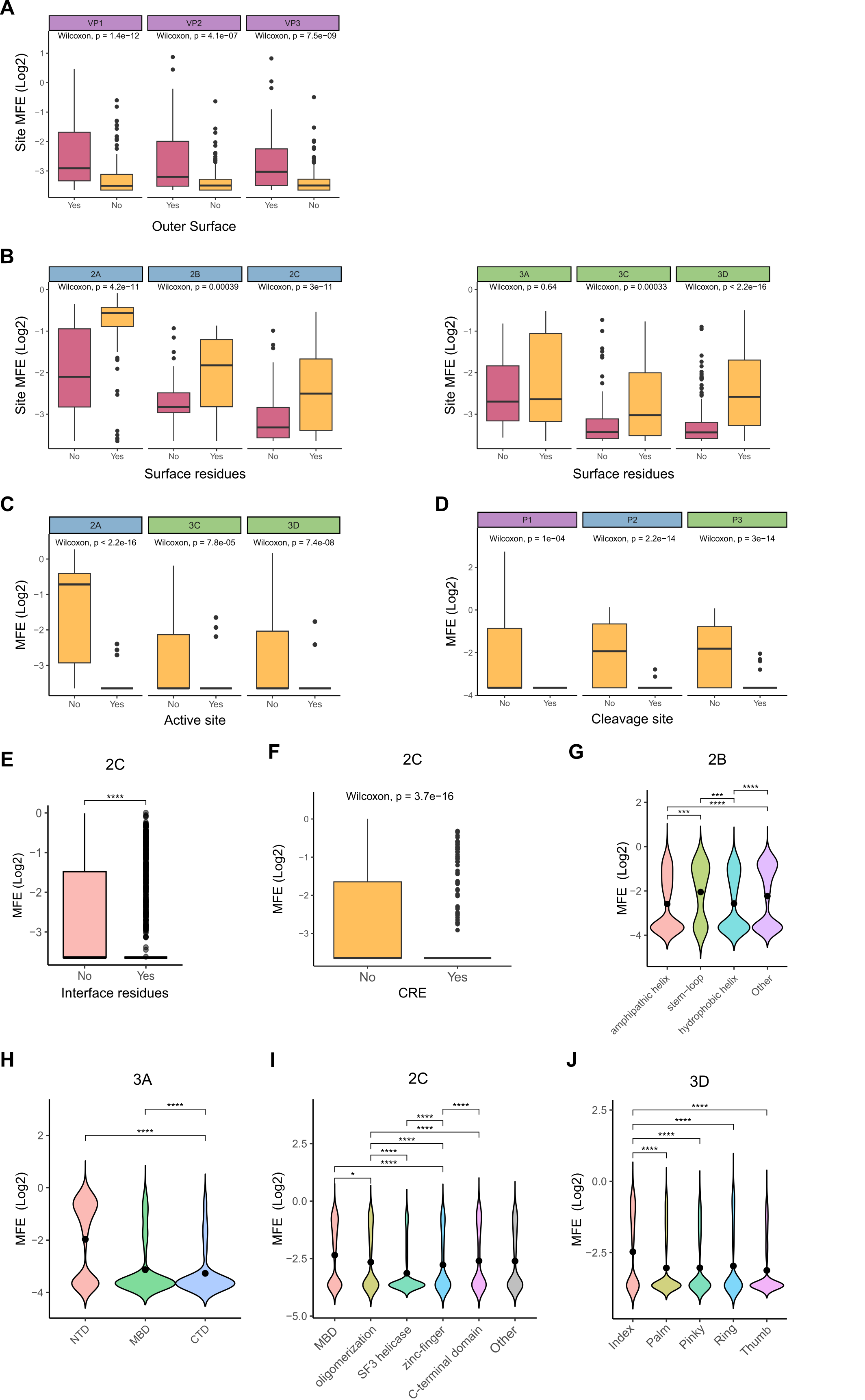

Supplement: S4 Fig — (A, B) Distribution of site MFE in outer surface residues versus other residues in the capsid proteins VP1-VP3 (A) and in surface-exposed residues versus internal residues for the nonstructural proteins (B). (C, D) Distribution of MFE in the active sites of the 2A and 3C proteases, and the 3D polymerase versus all other residues in each protein (C), and in the 3C protease cleavage site Q residues of each protein versus all other Q residues in that same protein (D). (E) Distribution of MFE in interface residues between monomers versus other residues in 2C. (F) Distribution of MFE in the CRE element versus other residues in 2C. (G–J) Distribution of MFE in the different structural and functional domains of the 2B (G), 3A (H), 2C (I), and 3D (J) proteins. ns: p > 0.05, *p < 0.05, **p < 0.01, ***p < 0.001, ****p < 0.0001 by Mann–Whitney test following multiple test correction. The data underlying this figure can be found in S1 Data, pages 11–20. (TIFF) [file pbio.3002709.s004.tiff]

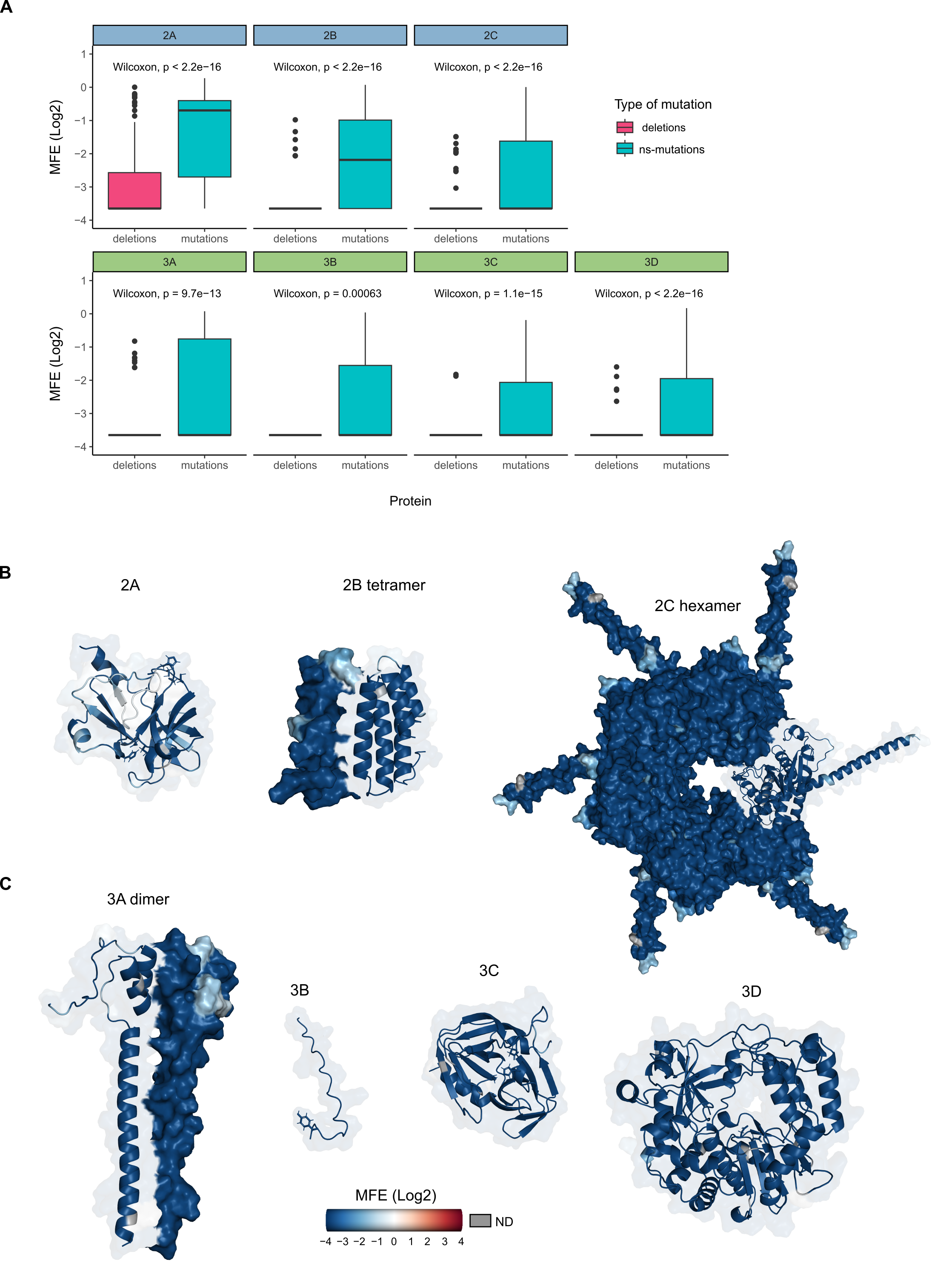

Supplement: S5 Fig — (A) The effects of non-synonymous mutations (ns-mutations) versus deletions across the nonstructural proteins. (B, C) Mapping of dMFE in P2 (B) and P3 (C) derived proteins. The data underlying this figure can be found in S1 Data, page 21. (TIFF) [file pbio.3002709.s005.tiff]

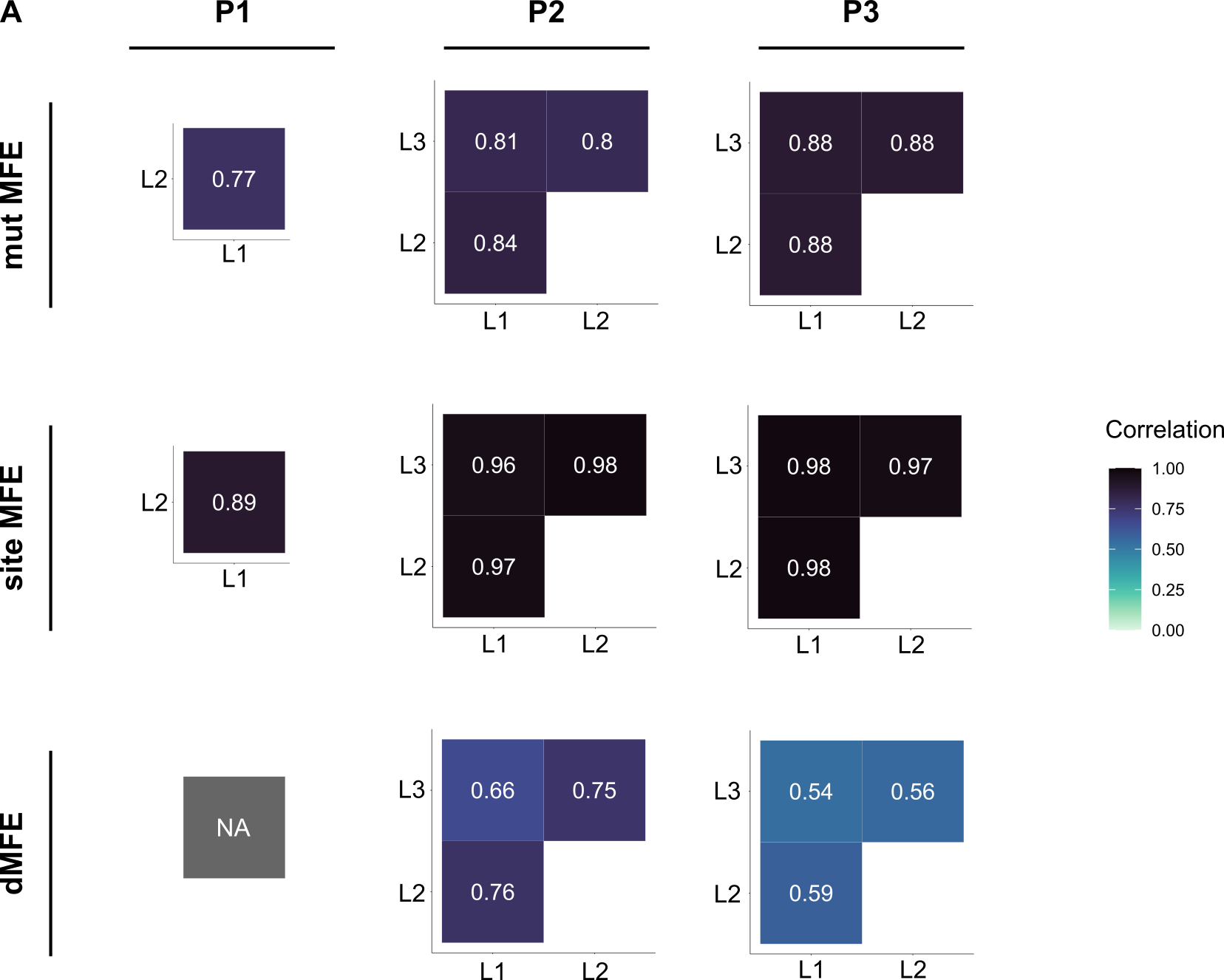

Supplement: S6 Fig — (A) Correlations matrices for MFE of mutations (mut MFE), their average per site (site MFE), and deletions (dMFE) for independent replicate lines (L1-L3) for the P1, P2, and P3 regions. Of note, for P1, only 2 replicates were used and deletions were not included in the mutagenesis protocol, precluding their analysis. (TIFF) [file pbio.3002709.s006.tiff]

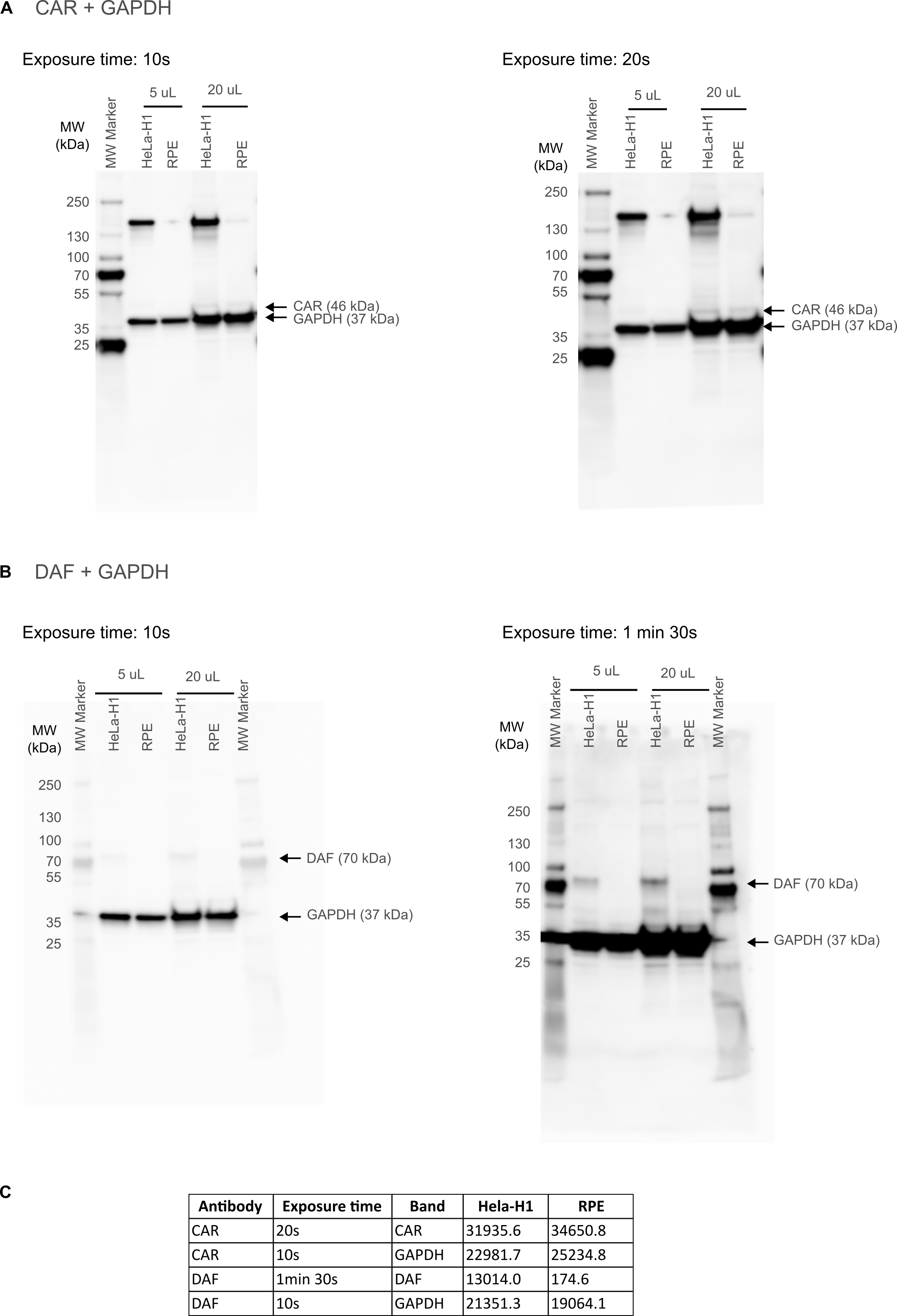

Supplement: S7 Fig — (A) The complete membrane is shown for western blots of CAR (A) and DAF (B) in both cell lines. Arrows indicate bands of the expected size for each protein. A higher molecular weight cross-reactive band is observed for CAR in HeLa-H1 cells. (C) Values obtained for the quantification of protein bands. Exposure times of blots used for each protein are indicated. (TIFF) [file pbio.3002709.s007.tiff]
