# Supplementary material for: Mapping mutational fitness effects across the coxsackievirus B3 proteome reveals distinct profiles of mutation tolerability
Source: PLoS Biol. 2024 Jul 16;22(7):e3002709. doi: 10.1371/journal.pbio.3002709 (PMC11251597; doi:10.1371/journal.pbio.3002709)

Figure S7 Raw, uncropped gels

A CAR + GAPDH

Exposure time: 10s

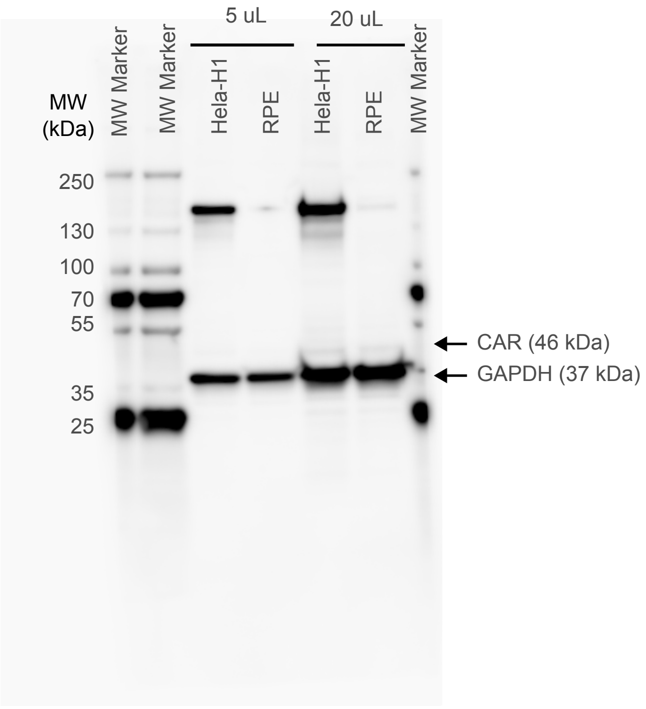

Exposure time: 20s

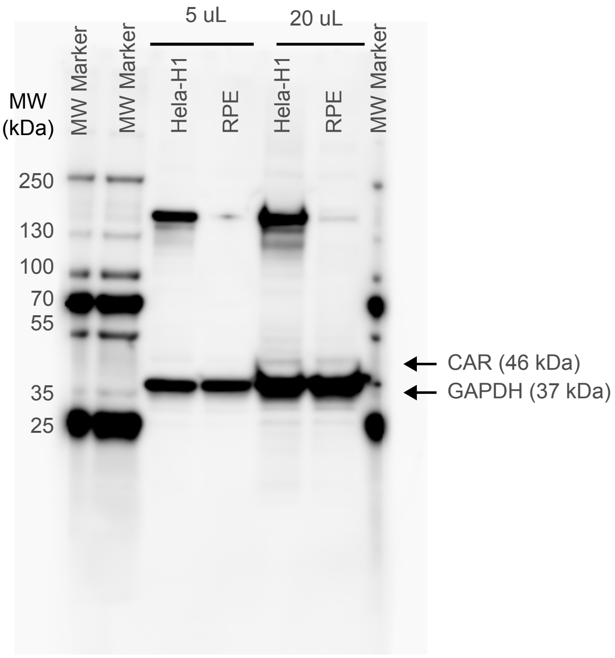

B DAF + GAPDH

Exposure time: 10s

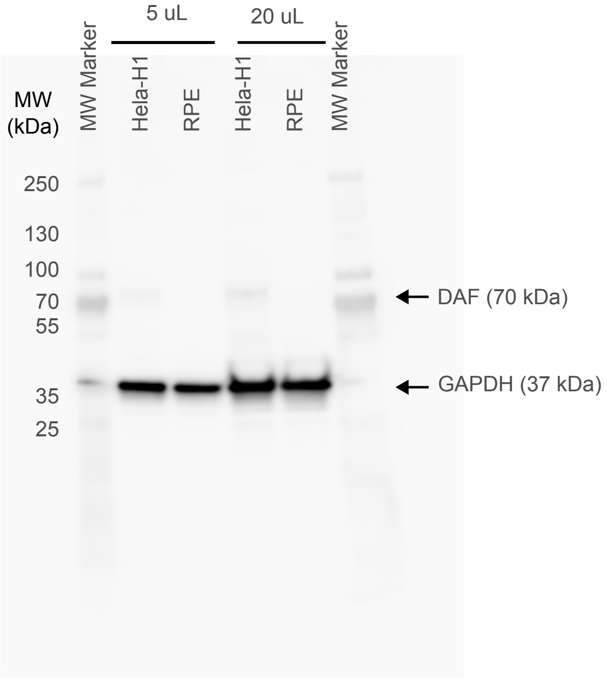

Exposure time: 1 min 30s

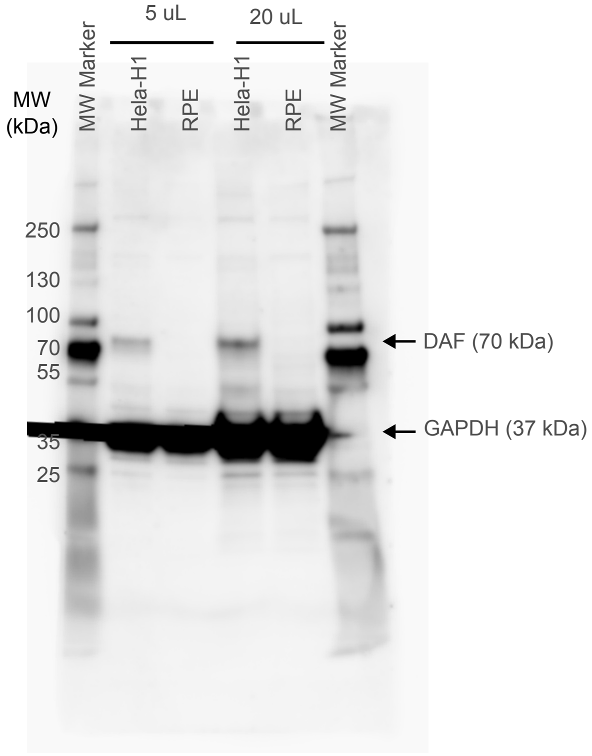

Supplement: S1 Raw Images — (PDF) [file pbio.3002709.s017.pdf]
