# Supplementary material for: Mapping mutational fitness effects across the coxsackievirus B3 proteome reveals distinct profiles of mutation tolerability
Source: PLoS Biol. 2024 Jul 16;22(7):e3002709. doi: 10.1371/journal.pbio.3002709 (PMC11251597; doi:10.1371/journal.pbio.3002709)
Supplement: Attachment — Submitted filename: response_to_reviewers_vs3.pdf [file pbio.3002709.s018.pdf]

Point-by-point response to reviewer's comments:

We would like to thank all the Reviewers for being positive about the results and their helpful comments. Below we have provided answers to all comments raised by the reviewers in blue text.

REVIEWERS' COMMENTS

Reviewer #1:

This is a really excellent deep mutational scanning study of coxsackievirus B3. It is comprehensive, well done, and clearly described. As the authors note, these results are interesting both from the basic perspective of understanding this virus and its proteins, and from the applied perspective of drug design.

I strongly support publication, and only have a few minor comments listed below.

- The authors assembled 250 nt oligos for mutagenesis, which I think (?) is a novel approach. I would have expected this to give a lot of errors in regions flanking the targeted codon, as typically oligo synthesis for very long (250 nt) oligos is quite error prone. However, as best I can tell from Fig S1, this does not seem to be the case. Can the authors provide more details? Perhaps Twist has gotten much more accurate in synthesis?

We have sequenced a large number of clones from the two regions where this method was used (n=240) and observe very few to have >1 codon mutation (n=16, 6.7%). Hence, empirically, the mutation rate is relatively low but not so far away from the expected error rate (240 clones x ~250 bp x 0.067 error rate = ~1 error in 4,000; the company claims an error rate of 1:3,000). One potential explanation for lower mutation rates in our data set could come from the fact that we amplify the ssDNA oligo pool with primers that bind to the termini of the long oligos ensuring that all PCR products contain the correct termini for HiFi DNA assembly. This should minimize any errors in these complementary regions and avoid the amplification and cloning of incomplete synthetic oligonucleotides into the infectious clone.

In terms of the novelty of using synthetic oligos for doing DMS, we know of at least 1 article that has used a similar synthetic approach (10.1126/science.aaw2900).

- Lines 157-159: the text indicates the averaging across replicates was done on the ratios, but was it actually done on the log ratios? If not, maybe it should be averaged on log ratios rather than ratios since the former are what is reported in the paper.

We apologize for not making this clear. The average was done on the log2 ratios. This has been clarified in the main text and the methods for the calculation of MFEs (lines 159 and 753) and differential selection (lines 404, 407 and 773).

- The correlations between replicates were quite good (Fig S2) for mutations, but are rather poorer for deletions. Why is this?

The correlations between replicates for the deletions are worse than the ones for non-synonymous mutations because most deletions are lethal. Lethal mutations are represented by the presence of a count in the sequencing data pre-selection, and the absence of counts in the sequencing of the viral populations (post-selection). The bioinformatic pipeline adds a pseudocount of 1 to all mutation counts in order to avoid having a zero in the numerator. This pseudocount is scaled up for the sample with higher depth. Hence, the MFE values for lethal mutations are strongly conditioned by the depth of the coverage, making them more variable than mutations where actual counts are observed in the viral populations. As a result, correlation between replicates of non-lethal (viable) deletions are similar or higher than those of the full dataset, despite very few datapoints in the former (see figure below). Similarly, the correlations for deletions in P2 is higher than for P3, likely because there is a higher number (~5x more) of non-lethal deletions in the P2 populations than in the P3 ones.

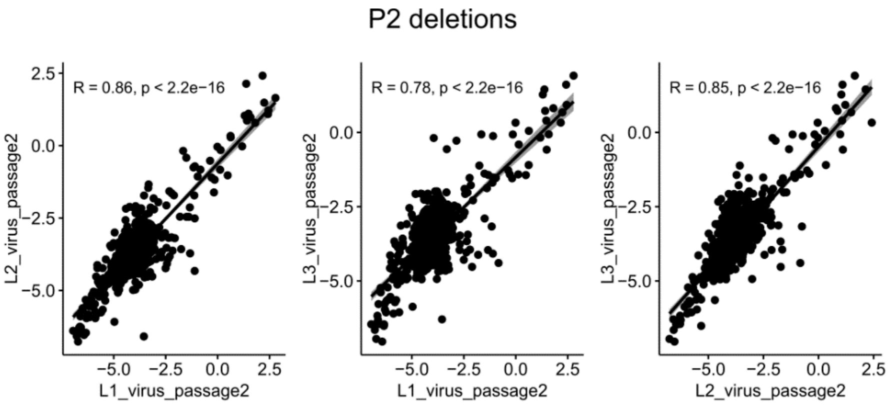

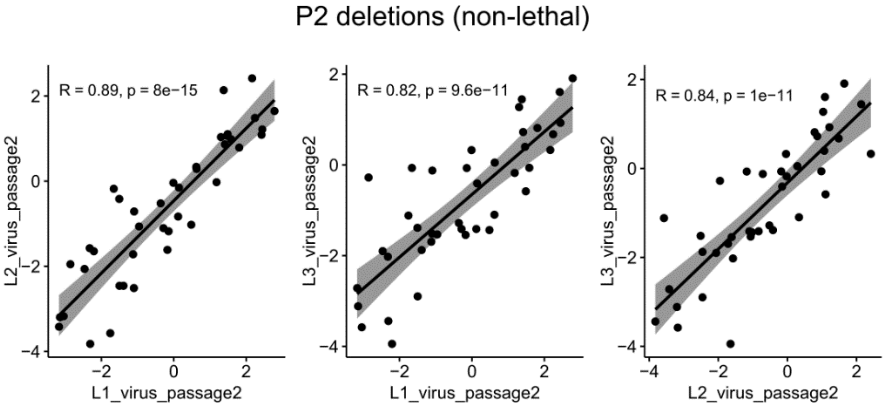

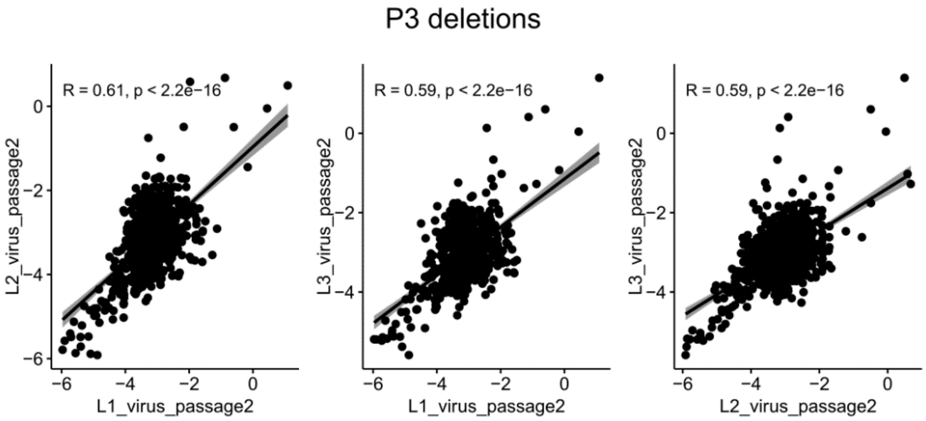

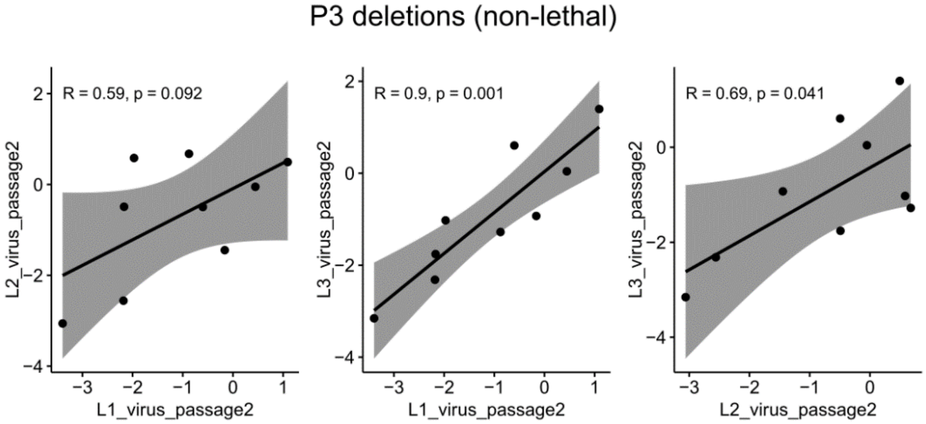

Reviewer #2:

This paper from Álvarez-Rodríguez and colleagues uses a synthetic biology-based deep mutational scanning (DMS) approach to compare mutational fitness effects (MFE) of amino acid substitutions and single codon deletions across the entire proteome of coxsackievirus B3. In doing this large-scale comparison, they can quantify how patterns of relative mutational tolerance differ across proteins encoded by the same virus. This is a fascinating question with significant implications for our understanding of viral evolution.

The authors detail significant differences in mutational tolerance based on specific protein, WT and mutant amino acid, and structural context. Interestingly, they find that the 2A protease is more mutation tolerant than the rest of the viral proteome, while the 3C protease is much less tolerant. They go on to show that MFE profiles are largely similar between viral populations grown in two distinct cell types, with the exception of residues involved in interacting with a viral receptor not present in one of the cell types. Finally, they examine mutational tolerance in 12 "druggable pockets" predicted through structural analyses and

detail how these pockets differ in tolerance profiles, with the assumption being that these differences could be used to predict probability of escape from drugs that target these specific pockets.

Overall, the paper is clearly written, the methods are generally sound (with a couple concerns detailed below) and the conclusions are well supported by the data. Assuming these relatively minor concerns are adequately addressed, I feel this paper will be a significant contribution to the field of viral molecular evolution.

Specific Concerns:

1. While the authors clearly describe their reasonable approach to normalizing fitness effects across the three regions of the viral genome that were experimentally analyzed separately, I think more is needed to further verify the absence of significant artifacts arising from combining data generated from three separate viral libraries/passage experiments. First, it looks like the authors introduced nonsense and synonymous substitutions into the genome. Assuming stop codons will be lethal and silent substitutions generally neutral, can they compare the relative fitness distributions for stop codons and silent substitutions across P1, P2, and P3, before and after linear model-based normalization, to assess how similar they are?

The MFE value of stop codons and any other lethal change is highly dependent on the coverage of each position, as explained above for deletions. Thus, lethal changes have been excluded from the fitting of the linear models and cannot be used to assess the precision of the correction.

As suggested by the Reviewer, while synonymous changes are not included in the linear models, they can be used to validate the normalization process of the three genomic regions. The median value of synonymous substitutions in the raw DMS datasets (on a log2 scale) are: -1.12, 1.13, and 1.8 for P1, P2, and P3, respectively (see graph below, left panel). Hence, a clear deviation from neutrality (MFE=0) is observed across the different regions in the raw data. This deviation may be explained by differences in the amount of actual WT virus present in each mutagenized population and/or differences in the ability of the proteins encoded in each region to tolerate mutations.

Specifically, MFE are calculated by examining the change in the relative frequency of a mutation versus the WT codon (count of a mutant codon divided by the count of the WT codon at a given position) following selection for growth in cells compared to the relative frequency in the plasmid libraries. The complication arises because the counts of the WT codon reflect both real WT viruses/plasmids (without any additional mutations) and mutant viruses/plasmids bearing mutations at other positions. As a result, under selection, the count of the WT codon at any given position reflects the average population fitness of both WT

virus and all mutant viruses having a mutation outside of this position. Since most mutations are deleterious, the average fitness of the populations is therefore lower than the real WT virus fitness, although this will depend on how much actual WT virus is present and how deleterious mutants are in a given region.

While this does not affect the relative differences between MFE obtained in the same experiments, it makes it more difficult to compare MFE across experiments and/or regions and to match the DMS-derived MFE with the actual fitness of the WT virus, necessitating some sort of correction between different libraries. Indeed, in our experiments, the Sanger sequencing analysis reveals different amounts of WT virus in each population (Figure S1; larger fraction of WT virus in the P1 DMS), and we also see different tolerance to mutations in the three regions. Both of these can potentially account for the differences observed in the median MFE of synonymous changes between regions.

The correction based on the overlap region re-scales the P1 and P3 regions to P2 and, as expected, synonymous changes under this correction are more similar to each other (median log2 MFE of synonymous mutations: 1.40, 1.13, and 1.15 for P1, P2, and P3, respectively; see middle panel of figure). However, these values are still not close to neutral. Finally, correction of MFE according to the experimental fitness values of **non-synonymous** mutations brings these values closer to the expected neutral effects of synonymous mutations (-0.33, -0.48, and -0.47, respectively. See third panel of figure).

Altogether, this indicates that both corrections make biological sense, particularly considering that synonymous mutations were not taken into account when doing the corrections yet approach neutrality after the correction. Finally, additional variation that would justify correction between regions can stem from the fact that the experiments were done at different times and are therefore subject to variation from experimental conditions (e.g. differences in the passage number of the cells, etc.).

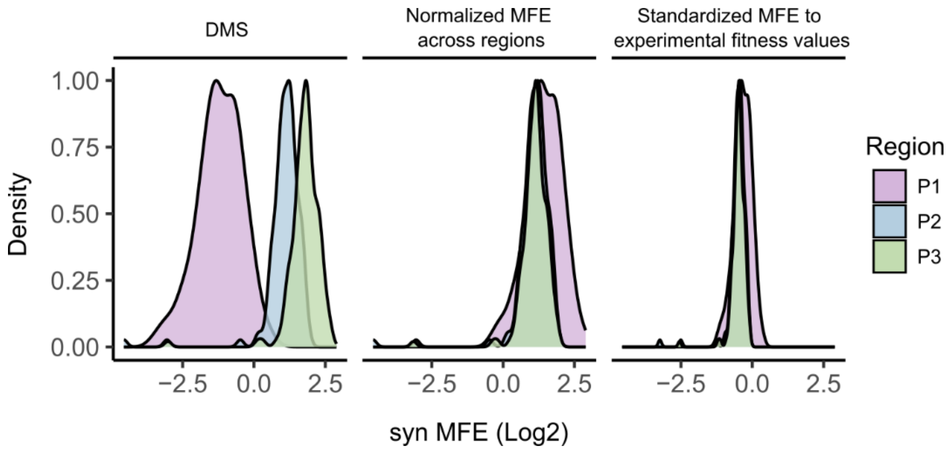

Also, is it possible to demonstrate how insensitive their findings are to variance in the linear models used to combine the three fragments?

The overall trends in MFE across proteins are similar before (raw DMS data) and after applying each standardization method (see below), with the major changes being the values of the MFEs, which reflect a more realistic fitness measurement relative to a real WT virus. The main conclusions of the work are consistent in all three cases, with the P1 region presenting the most deleterious but also the most tolerant sites, and 2A and 3A being the regions of the proteome with the highest mutational tolerance.

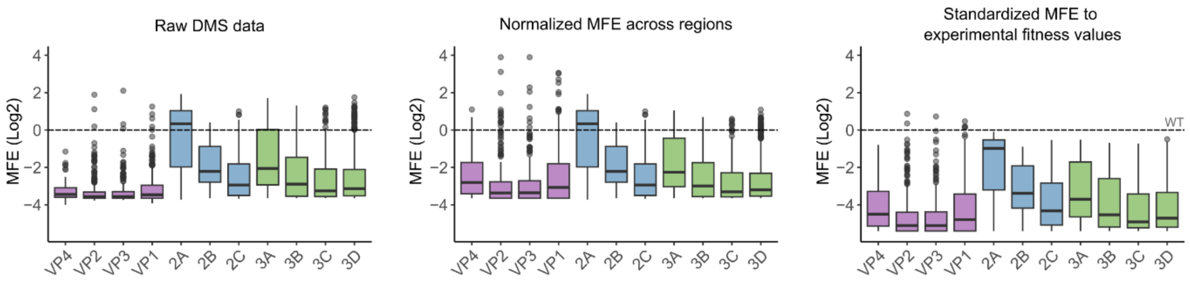

There are two additional observations that support the standardization of the three regions. The first one is that synonymous changes, which are expected to be neutral, get normalized to similar values, and closer to the expected value of WT, when the standardization is applied (see previous answer). The second observation that further validates the normalization across the three regions is that the correlation of the overlap-corrected dataset (Pearson's R = 0.78 p-value = 4.9 x 10^{-7}; see right panel below) is better than the correlation of the raw DMS dataset (Pearson's R = 0.62, p-value = 2.6 x 10^{-5}; see left panel below) with the values obtained in the experimental validation (see figure below). Thus, an independent experimental measurement further supports the normalization applied across regions. In summary, all these observations support that the standardization calibrates the three regions to a more meaningful scale, without changing the overall message of our findings.

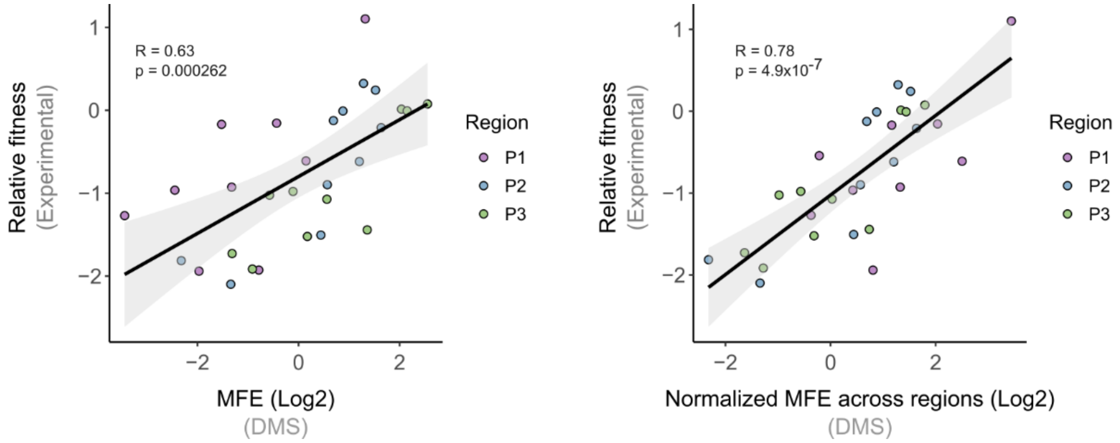

Finally, can the authors show the outliers form their comparison of experimental and DMS-derived relative fitness estimates? Is there any reason to believe there could be systematic or biological explanation for divergence in these two measurements of relative fitness that could explain the outliers?

Two datapoints were removed from the analysis as outliers and this did not impose a significant change to the linear model obtained [see figure below representing the fitting of the DMS dataset to the experimental dataset with (left panel) or without (right panel) outliers and their corresponding linear models]. These outliers can be explained by the different sensitivity of the DMS assay versus the experimental competition assay, and /or by the fact that bulk assays such as DMS contain a certain level of error. The full dataset of the competition assay results and the code to obtain the linear models with and without outliers is available in Github (section B4), which is referenced in the legend of Figure 2 (line 191) and the methods section (line 762), and thus is readily available to any interested reader.

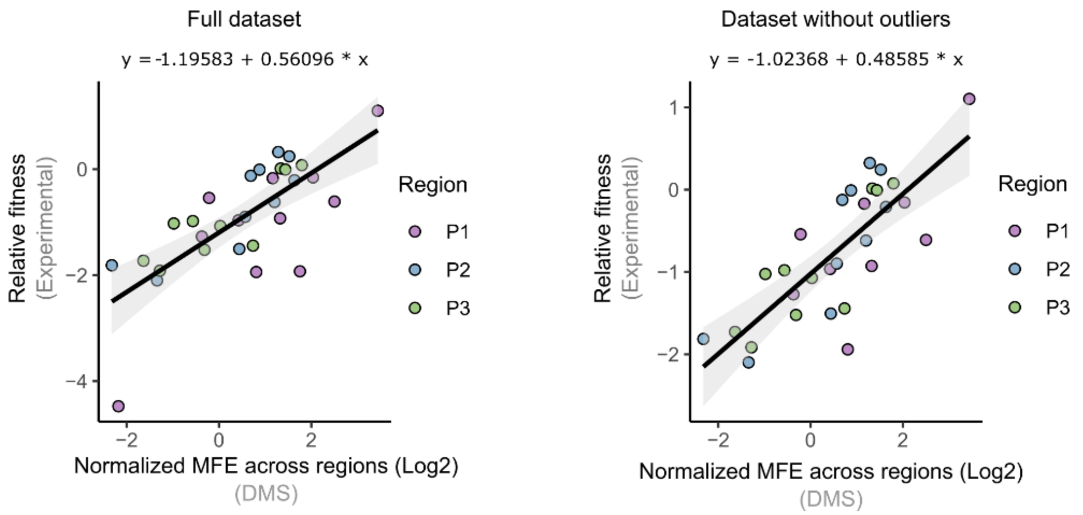

2. In the cell type comparison experiment, the infections in RPE cells were conducted at MOI = 2 (line 694), which is significantly higher than it was in HeLa-H1 and can potentially have a complementary and/or genotype masking effect due to the high probability of coinfection on the population thus confounding the comparison. The authors should either repeat this experiment using equivalent low MOIs in both cell lines.

We apologize for not making this clear in the text. The titer indicated in the methods corresponds to a titration in HeLa-H1 cells, not RPE. As indicated in the main text, RPE are more restrictive (116 ± 13-fold infectivity reduction), and thus a higher virus titer was used to infect RPE cells in order to compensate for their lower infectivity. Therefore, both experiments were done using similar effective MOI of 0.1. We have added this information to the methods section (lines 702-704).

3. Since RPE cells have a stronger interferon response than HeLa-H1, is it surprising that the authors did not observe differences in mutational tolerance in regions known to be involved in counteracting the IFN response?

Indeed, RPE cells present a stronger type I interferon response than HeLa-H1 when pre-treated with IFN-I beta prior to infection. However, our experiments were performed in the absence of any IFN-I treatment. How the different cell lines mount innate immune responses upon infection by CVB3 in the absence of IFN-I pre-treatment was not defined in this work. Both scenarios are being studied in our lab now using the DMS populations as part of a separate project and we believe it is outside of the scope of the current work.

4. Line 212, maybe specify a little more here on the sequence set used rather than just having details in methods. Wasn't clear how many total sequences across how many years were included in final analysis.

We apologize for not including this information before. We have now indicated both the number of sequences and the range of collection dates in the text (lines 215-216), and included a description of all samples in the GitHub repository.

5. In figure S1A, please confirm that the number of changes per clone refers to amino acid substitutions. Also, if these are amino acid substitutions, why do so many clones in P1 (where mutations were introduced through PCR) have more than 1 amino acid substitution? I assume that in P2 and P3, multiple mutation containing tiles can get combined into a single haplotype during the PCR process? I may have misunderstood the library construction method, so clarification would be appreciated here.

The number of changes in this figure refers to number of codons altered per clone, either involving one, two or three substitutions per codon, and including synonymous codon changes (this has now been added to the figure legend of Figure S1). The PCR method used for the P1 region can result in more than one codon mutated per clone as it follows a Poisson distribution, as has been observed in multiple publications from Jesse Bloom's lab (e.g. 10.1093/molbev/msu173). In the case of the synthetic oligonucleotides approach (P2 and P3) each tile is cloned separately into a PCR of the remaining (WT) infectious clone sequence, thus multiple codon mutations stemming from the combination of more than one tile are not possible. Rather, multiple codon mutations in these clones (within P2 and P3) could arise from errors in the oligonucleotide synthesis process (estimated to present an error rate of 1:3,000 nucleotides by the synthetizing company), errors in the PCR amplification step to generate dsDNA products from the ssDNA oligonucleotides or errors in the seamless cloning of the tiles into the vector.

Reviewer #3:

Álvarez-Rodríguez et al. perform systematic mutagenesis together with a prior dataset from the group that details the complete mutational landscape of the coxsackievirus B3 proteome. The scale of experiments is impressive, and the authors draw out a number of patterns in the mutagenesis data relative to structural and evolutionary correlates to make sense of these data. The authors also identify variation in mutational tolerance of druggable pockets, illustrating the utility of prospective mutational profiling in selection of potential drug targets with less capacity for evolution of resistance. Overall, the study is well performed and analyzed, and I have just a few minor comments.

Intro highlights the different proteins of CVB3, but then first section of results start discussing "P1", "P2", "P3". It could be quickly included either in the intro or when first mentioning P1-3 how these map to the sets of structural/non-structural proteins themselves.

Thank you for pointing this out. We apologize for not including this initially and have added this information to the main text (lines 83 and 86).

Most of the structural and evolutionary correlates of mutational tolerance make good sense, but Fig. 2C is difficult to understand how it can show that, for example, mutations to D is maximally deleterious in every instance. It wouldn't be surprising to see some D mutations be deleterious at particular types of amino acid positions, but I wouldn't expect every single mutation to D to be so deleterious at every position tested. (My assumption was this was a box-and-whiskers plots across all measured mutants of different wildtype or mutant classes.) Even mutations like P, G, etc. which are 'unique' amino acids, I wouldn't expect there to be universal detrimental effect at every single position, but especially for a more common amino acid like D, I find it hard to understand that all mutations to D are deleterious. In fact the heatmap in Fig. 2B shows plenty of 'white' scattered through the strip of D mutants, therefore it's not clear why they are absent in the box and whiskers plot. Am I interpreting Fig. 2C correctly? I feel like there must be an error here.

The reason for the apparent discrepancy is that we have omitted the outliers in the boxplots to increase clarity, which includes all values that are outside the 1.5*interquartile range. Hence, there are cases where the mutations are tolerated but these are not reflected in the boxplot. We apologize for not making this clear in the text, and now have added this information to the figure legend (lines 183-185).

Was any analysis done with respect to synonymous mutations with unexpected deleterious effects that could point to interesting constraints on underlying nucleic acid structures? Though I see in the Methods that synonymous mutations were not routinely included since full NNN mutagenesis was not performed.

As the Reviewer indicates, we have not systematically introduced synonymous mutations in the non-structural regions (P2 and P3), and in P1 (~1/3 of the proteome), despite using an NNN approach, we omitted all single changes per codon, which removes many silent

mutations. In total, we have sampled ~18% of possible synonymous mutations, with nearly all being single codon substitution per residue, which could end up not affecting secondary structure. We do observe deleterious effects for silent mutations at known RNA structures (e.g. CRE in 2C and the 3' of 3D; see below). We prefer to not go into this in detail in the current work as we do not feel we have done an exhaustive analysis of silent mutations. That said, both the data and the scripts for looking at synonymous mutations are available on the project's GitHub.

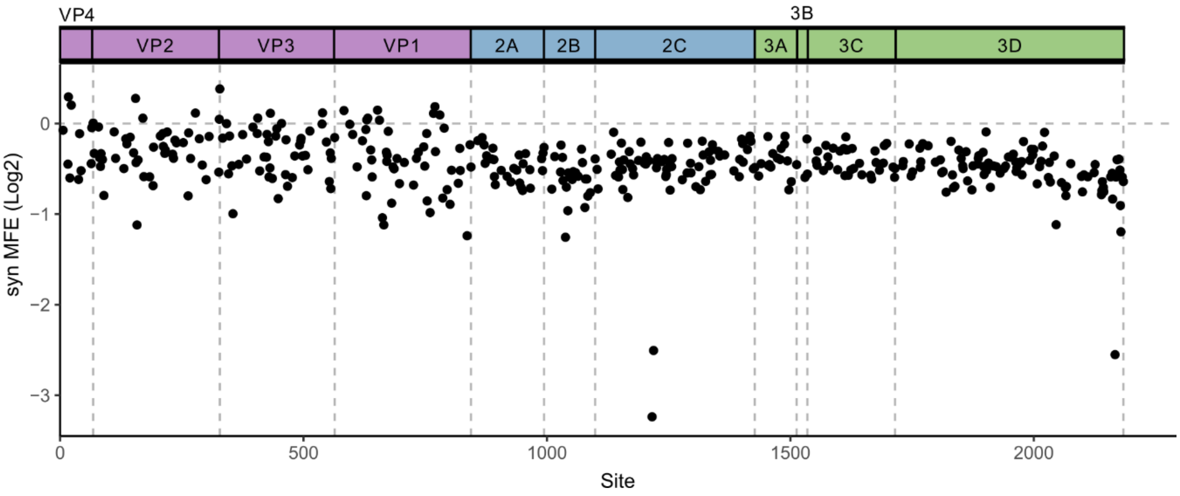

Main text could describe what software or method is used to predict druggable pockets, or at least give intuition for what the Schrodinger software is looking for when it predicts pockets.

We thank the Reviewer for their suggestion and have included in the Main text details on the software and parameters used for the drug pocket prediction (lines 440-445).
